# Chronic Low-Dose-Rate Radiation-Induced Persistent DNA Damage and miRNA/mRNA Expression Changes in Mouse Hippocampus and Blood

**DOI:** 10.3390/cells13201705

**Published:** 2024-10-15

**Authors:** Hong Wang, Salihah Lau, Amanda Tan, Feng Ru Tang

**Affiliations:** Radiation Physiology Laboratory, Singapore Nuclear Research and Safety Initiative, National University of Singapore, Singapore; snrwh@nus.edu.sg (H.W.); salihahl@nus.edu.sg (S.L.); amanda97@nus.edu.sg (A.T.)

**Keywords:** low-dose-rate, chronic irradiation, neonatal, brain, blood, cellular, miRNA and mRNA

## Abstract

Our previous study demonstrated that the acute high-dose-rate (3.3 Gy/min) γ-ray irradiation (γ-irradiation) of postnatal day-3 (P3) mice with 5 Gy induced depression and drastic neuropathological changes in the dentate gyrus of the hippocampus of adult mice. The present study investigated the effects of chronic low-dose-rate (1.2 mGy/h) γ-irradiation from P3 to P180 with a cumulative dose of 5 Gy on animal behaviour, hippocampal cellular change, and miRNA and mRNA expression in the hippocampus and blood in female mice. The radiation exposure did not significantly affect the animal’s body weight, and neuropsychiatric changes such as anxiety and depression were examined by neurobehavioural tests, including open field, light-dark box, elevated plus maze, tail suspension, and forced swim tests. Immunohistochemical staining did not detect any obvious loss of mature and immature neurons (NeuN and DCX) or any inflammatory glial response (IBA1, GFAP, and PDGFRα). Nevertheless, γH2AX foci in the stratum granulosum of the dentate gyrus were significantly increased, suggesting the chronic low-dose-rate irradiation induced persistent DNA damage foci in mice. miRNA sequencing and qRT-PCR indicated an increased expression of miR-448-3p and miR-361-5p but decreased expression of miR-193a-3p in the mouse hippocampus. Meanwhile, mRNA sequencing and qRT-PCR showed the changed expression of some genes, including *Fli1*, *Hs3st5*, and *Eif4ebp2*. Database searching by miRDB and TargetScan predicted that *Fli1* and *Hs3st5* are the targets of miR-448-3p, and *Eif4ebp2* is the target of miR-361-5p. miRNA/mRNA sequencing and qRT-PCR results in blood showed the increased expression of miR-6967-3p and the decreased expression of its target *S1pr5*. The interactions of these miRNAs and mRNAs may be related to the chronic low-dose-rate radiation-induced persistent DNA damage.

## 1. Introduction

It is widely accepted today that high dose/dose rate γ-ray irradiation (γ-irradiation) induces harmful effects on the central nervous system (CNS), especially on the developing brain, which is particularly vulnerable to the harmful impacts. Irradiation exposure during the prenatal period may have a broad spectrum of consequences, including congenital abnormalities, mental retardations, developmental delays, behavioural alterations, and functional deficits, varying depending on the different doses [1,2,3]. The germ cells in the testes and ovaries of mice were almost obliterated on gestation day 18 for foetuses irradiated with continuous medium-dose-rate (200 or 400 mGy/day) γ-rays throughout the entire gestation period [4]. Very recently, we found that prenatal irradiation of B6C3F1 mice (100 mGy/d for 18 days) did not induce cellular changes, including mature neurons and glial cells in the hilus of the dentate gyrus and immature neurons in the subgranular zone [5]. Substantial negative effects on the developing human brain have been observed among children exposed to radiation during the gestational period from the atomic explosions of Hiroshima and Nagasaki [6,7] as well as over the time of the Chernobyl and Fukushima accidents [1,8,9]. Long-term cytogenetic effects indicated by the prevalence of chromosome aberrations were observed in children prenatally exposed to the Chernobyl nuclear accident [10]. The effects of prenatal irradiation on the development of cerebral electrical activity were investigated in humans as early as 1968 [11]. Nevertheless, the consequences of postnatal irradiation exposure have not been extensively studied, especially after the low-dose-rate chronic γ-radiation exposure.

The development of the hippocampus plays an important role in its physical maturation. High-dose-rate (3.3 Gy/m) acute γ-irradiation (5 Gy) of day-3 mice has induced depression behaviour in adults accompanied by the hypoplasia of the infrapyramidal blade of the stratum granulosum as well as impaired neurogenesis and cell division in the dentate gyrus [12]. A very recent study indicated that a fractionated γ-radiation in neonatal C57BL/6 at cumulative doses (0.1, 1, and 5 Gy) caused mouse behaviour changes dose-dependently; the low-dose γ-irradiation resulted in increase in anxiety, while the raised dose caused a decrease in anxiety behaviour compared to control animals [13]. Moreover, Tanaka et al. reported that mice chronically exposed to low-dose-rate γ-rays from 8 weeks of age had significantly shorter life spans than non-irradiated mice [14] due to early death from a variety of neoplasms [15]. This evidence indicated that neonatal or adult γ-radiation in mice causes behavioural or cellular changes. Acute X-ray irradiation with 2 Gy at postnatal day 3 induced the impairment of spatial learning and memory and anxiety in adult mice, accompanied by increased levels of γH2A histone family member X (γH2AX) [16]. An extensive induction of γH2AX foci was observed in different brain regions at 1 day after 5 Gy γ-irradiation at postnatal days 3, 10, and 21 in mice, and lasted for 15 months after irradiation [17], suggesting that γH2AX serves as a marker for γ-radiation-induced DNA damage and is related to animal behaviour changes.

Our previous study with acute high-dose-rate irradiation of postnatal day-3 mice with 5 Gy induced obvious pathophysiological changes [12]. In the current study, we aimed to examine the effects of continuously chronic low-dose-rate γ-irradiation for 180 days with a cumulative dose of 5 Gy on animal neuropsychiatric changes. Relevant hippocampal cellular miRNA and mRNA changes were investigated. Meanwhile, blood miRNA and mRNA changes were also detected to correlate these changes to those in the hippocampus and explore the possibility of using blood miRNAs and mRNAs as biomarkers to predict low-dose-rate irradiation-induced brain pathophysiological changes.

## 2. Materials and Methods

### 2.1. Animals and Irradiation

Postnatal day-1 Balb/c pups with dams were purchased from InVivos Pte. Ltd. (Singapore) and housed in the Department of Comparative Medicine Facility, National University of Singapore. From postnatal day 3 (P3) onwards, pups with dams were continuously exposed to whole-body ^137^Cs γ-rays (23.5 h/daily exposure, 0.5 h for animal feeding and cage cleaning) with a dose rate of 1.2 mGy/h (G10-1-12 Gamma Beam Irradiator, Alpharetta, GA, USA). By postnatal day 21, pups were weaned, and male and female pups were separated and continuously exposed to radiation with a low dose rate of 1.2 mGy/h up to 180 days (a total accumulated dose of ~5 Gy). Mice were then moved to a normal mouse room with background radiation. The radiation dose rate was monitored during irradiation with a MAX 4000 Plus electrometer (Standard Image, Middleton, WI, USA). The accumulated dose was measured with Nanodots (LANDAUER, Glenwood, IL, USA), which were placed in each cage. The animal experimental protocols were approved by the Institutional Animal Care and Use Committee (IACUC), National University of Singapore (IACUC number: R20-0220). A total of 24 female mice, including age-matched non-irradiated controls (control: n = 9; irradiated group: n = 15), were used in the study. All animals had free access to food and water. They were maintained under the following conditions: a 12 h light/12 h dark cycle and constant temperature of 22 °C, weekly cage change, and daily health monitoring. Animal body weight was measured weekly for the first 4 weeks, biweekly for the next 8 weeks, then continuously monitored and weighed before mice were euthanized. Efforts were made to minimize the number of animals used throughout the study.

### 2.2. Behavioural Studies

Three hundred and fourteen days after irradiation, irradiated and age-matched control Balb/c female mice were tested in open field, light-dark box, elevated plus maze, tail suspension, and forced swim tests. 

#### 2.2.1. Open Field Test

The open field was performed as described previously [5]. Mice were placed in a 50 cm x 50 cm opaque box. The arena was divided into center and peripheral areas in the ANY-maze software (version 7.10., ANY-maze, Wood Dale, IL, USA), which detected the center of the mouse’s body. The mouse was placed in the center area at the start of the test and was allowed to explore for 30 min, and its behavior was observed. The time spent and the distance travelled in each area were recorded.

#### 2.2.2. Light Dark Box

The experimental apparatus consists of a 50 cm × 50 cm box divided into two halves, each measuring 50 cm × 25 cm, with one half designated as the light zone and the other as the dark zone. Mice were placed in the light box first and their behaviour was recorded for 10 min. The time spent in the light and dark box and the distance travelled in the light box were recorded by ANY-maze version 7.10. (ANY-maze, Wood Dale, IL, USA).

#### 2.2.3. Elevated Plus Maze

The maze was elevated to 50 cm in height. The size of the open and closed arms was 5 cm × 30 cm and the center was 5 cm × 5 cm. Animals were placed in one closed arm and recorded for 10 min. The time spent and the distance travelled in the open and closed arms and the center were calculated by ANY-maze version 7.10. (ANY-maze, Wood Dale, IL, USA).

#### 2.2.4. Tail Suspension Test

The tail suspension test was performed as described previously [5]. Mice were suspended by their tails, taped to a hook for 6 min. Immobility time, in terms of no limb movements for 3 s or more, was recorded by ANY-maze version 7.10. (ANY-maze, Wood Dale, IL, USA). Immobilization is used as an indication of depression-like behaviour.

#### 2.2.5. Forced Swim Test

A forced swim test was performed as described previously [5]. A cylinder of 20 cm diameter was filled with water (temperature: 24–26 °C). The mice were placed inside and swam freely for 8 min. Animal movements were recorded and analyzed using ANY-maze version 7.10. (ANY-maze, Wood Dale, IL, USA). Immobilization time (where the animal remained almost immobile without limb movements for 3 s or more) was used as an indication of depression-like behaviour.

### 2.3. Sample Collection

All female mice were sacrificed by carbon monoxide asphyxiation at 529 days of age. Blood samples were collected via cardiac puncture. A quantity of 0.5 mL whole blood was transferred to 1.3 mL RNAlater solution and stored frozen at −80 °C. The whole brain was dissected and separated sagittally into the left and right hemispheres. The left hippocampus was dissected from the left hemisphere and stored at −80 °C for RNA extraction. The right hemisphere was fixed in 4% paraformaldehyde for 24 h. Following fixation, the brains were transferred to a 30% sucrose solution in 0.1 M phosphate buffer (pH: 7.4) for immunohistochemical analysis.

### 2.4. Immunohistochemical Staining

A Leica CM1950 cryostat (Leica Biosystems, Nussloch, Germany) was used to cut the right hemisphere of mice sagittally at 40 μm thickness. A series of alternative 6 sections were collected in a 24-well-plate with PBS. Six series were assigned to 6 different antibodies. After being treated with 3% H_2_O_2_ and blocked with serum, free-floating sections were incubated with antibodies overnight. Six antibodies were used in immunostaining, namely newly generated neuronal marker doublecortin (DCX, 1:500, Santa Cruz Biotechnology Inc., Santa Cruz, CA, USA), mature neuronal marker NeuN (1:1000, Invitrogen, MA, USA), oligodendrocyte precursor cell marker platelet-derived growth factor receptor alpha (PDGFRα, 1:200, Cell Signaling Technology, Danvers, MA, USA), astrocyte marker glial fibrillary acidic protein (GFAP, 1:200, Cell Signaling Technology, Danvers, MA, USA), microglial marker ionized calcium-binding adapter molecule 1 (IBA1, 1:200, Cell Signaling Technology, Danvers, MA, USA), and DNA damage and repair marker γH2A histone family member X (γH2AX,1:200, Cell Signaling Technology, Danvers, MA, USA). The sections were washed two times with PBS-Triton X-100 (PBST) and then incubated with respective secondary antibodies. Avidin–biotin complex (ABC) reagent (Vector Laboratories Inc., Burlingame, CA, USA) was applied to the sections and incubated for 30 min. Following incubation, the samples were washed twice with PBST. Subsequently, DAB Peroxidase Substrate (Vector Laboratories Inc., Burlingame, CA, USA) was added to the sections, which were then washed again, mounted on slides, counterstained with hematoxylin, and covered with coverslips.

Eight control and seven experimental mouse samples were used for IHC examination. Seven to nine immunostained sections from each animal were photographed under microscopy (Leica Microsystems GmbH, Wetzlar, Germany). The Stereologer System (Stereology Resource Center, Biosciences Inc., Tampa, FL, USA) was employed to unbiasedly analyze the number of NeuN, PDGFRα, and GFAP immunopositive cells in the hilus and IBA1 immunopositive cells in the hilus and stratum granulosum, indicated as the number/volume (mm^3^). The volume was calculated by the software (Stereology™). As one of every alternative 6 sections was used for each antibody, the z-axis interval between sections for each antibody was fixed at 6 × 40 um = 240 um. The unbiased counting frame with two inclusion lines and two exclusion lines within the delineated area was set up to provide a rule for which cells should be counted; a cell was counted if it was inside the counting frame or lay on an inclusion line but not an exclusion line; the cells crossing the exclusion line were not counted. γH2AX foci in all the cells in the stratum granulosum were counted and indicated as the total number/area (mm^2^). DCX immunopositive cells were counted in the subgranular zone and showed as the number/per subgranular length (mm).

### 2.5. RNA Extraction from the Hippocampus and Whole Blood

RNA extraction from the hippocampus and whole blood was described previously [5]. Briefly, RNA extraction from the left hippocampus of 3 non-irradiated control and 3 irradiated mice was performed using the miRNeasy Mini Kit (Qiagen, Hilden, Germany) according to the manufacturer’s instructions. The hippocampus was homogenized in 700 µL QIAzol lysis reagent and allowed to rest for 5 min at room temperature. Following this, 140 µL chloroform was added to the tube, which was shaken vigorously for 15 s and then centrifuged at 12,000× *g* for 15 min at 4 °C. After centrifugation, the samples were separated into 3 phases. The upper colourless aqueous RNA phase was collected and mixed with 1.5 volumes of 100% ethanol. This mixture was loaded into a RNeasy Mini spin column and centrifuged at ≥8000× *g* for 15 s. After centrifugation, the column was washed and RNA was eluted from the column membrane with 40 µL RNase-free water.

RNA was isolated from whole blood using a Mouse RiboPure™-Blood RNA Isolation Kit (Life Technologies Holdings Pte Ltd., Singapore). Blood samples preserved in pre-loaded RNAlater solution were centrifuged, and the supernatant was discarded. The resulting cell pellet was reconstituted with a lysis solution containing 3 M sodium acetate and acid phenol:chloroform). After reconstitution, the cell pellet was centrifuged, and the upper phase was collected. One-hundred percent ethanol was added to this aqueous phase, and the mixture was subsequently vacuum-filtered through a Filter Cartridge. RNA was then washed and eluted with nuclease-free water.

### 2.6. Systematic miRNA Sequencing (miRSeq) and mRNA Sequencing Analysis

miRSeq and mRNA sequencing of the hippocampus and the blood were performed using the DNB SEQ platform (BGI, Beijing, China), which was described previously [5].

For mRNA sequencing, a certain amount of RNA samples was denatured and enriched by oligo (dT)-attached magnetic beads and fragmented. The first-strand synthesis reaction system was set up to synthesize the first-strand cDNA. The second-strand synthesis reaction system (including dUTP) was prepared to synthesize the second-strand cDNA. Double-stranded cDNA fragments were subjected to end-repair. Further, 3′ ends of the blunt fragments were added with a single ‘A’ nucleotide. Adaptor ligation was subsequently performed on the cDNAs, which were then amplified by PCR. Single-stranded cyclized products were produced and replicated via rolling cycle amplification. DNA nanoball (DNB), which contained multiple copies of DNA, was generated, loaded into patterned nanoarrays, and sequenced through combinatorial Probe-Anchor Synthesis (cPAS).

For miRNA sequencing, a certain amount of RNA sample was combined with a 3′ adapter, incubated, and then combined with a 5′ adapter. RT-PCR was performed. The products were purified with PAGE gel and dissolved in EB solution. Single-stranded circular DNA molecules were replicated via amplification, and DNA nanoball (DNB) that contained multiple copies of DNA was generated, loaded into patterned nanoarrays, and sequenced through combinatorial Probe-Anchor Synthesis (cPAS).

Based upon a *p*-value less than 0.05 and fold change of more than 1.5 between control and irradiated samples analyzed by the DESeq2 method, 140 and 186 differentially expressed miRNAs in the hippocampus and blood were detected, respectively. Similarly, 107 and 462 differentially expressed mRNAs were detected in the hippocampus and blood, respectively.

### 2.7. Real-Time Quantitative Reverse Transcription PCR (qRT-PCR) Analysis of mRNA

RNA was reverse transcribed into cDNA using Maxima first strand cDNA synthesis kits (Thermo Fisher Scientific, Waltham, MA, USA). A 20 µL reaction included 2 µL Maxima Enzyme Mix, 4 µL 5× Reaction Mix, 1 µg RNA, and nuclease-free water. The mixture was incubated at 25 °C for 10 min, 50 °C for 45 min, and 85 °C for 5 min.

A PCR master mix of 20 µL was mixed as follows: 10 µL 2× Maxima SYBR Green qPCR Master Mix, 4 µL nuclease-free water, 2 µL diluted cDNA, and 2 µL 10× forward and reverse primers for target genes (Table 1). PCR amplification was performed using QuantStudio 6 Real-Time PCR Systems (Thermo Fisher Scientific, Waltham, MA, USA). The mixture was denatured at 95 °C for 10 min, followed by 40 cycles of 95 °C for 15 s, 60 °C for 30 s, and 72 °C for 30 s. The expression of GAPDH was used as an internal control.

### 2.8. Real-Time RT-PCR Analysis for miRNA

RNA was reversely transcribed into cDNA using a miScript II RT kit (Qiagen, Hilden, Germany). A 20 µL reaction mixture contained 5 µL template RNA, 2 µL reverse transcripts mix, 4 µL 5× HiSpec buffer, 2 µL 10× nucleotide mix, and 7 µL nuclease-free water. The mixture was incubated at 37 °C for 1 h and then 95 °C for 5 min.

For real-time PCR, 20 µL of reaction mixture was prepared as 10 µL 2× miScript SYBR green PCR master mix, 4 µL nuclease-free water, 2 µL diluted cDNA, 2 µL 10× miScript universal primer, and 2 µL primer for target miRNAs (Table 2). PCR reactions were denatured at 95 °C for 15 min and then 40 cycles of 94 °C for 15 s, 55 °C for 30 s, and 70 °C for 30 s. QuantStudio 6 Real-Time PCR Systems (Thermo Fisher Scientific, Waltham, MA, USA) was used to perform PCR amplification and fluorescence data collection. miR-68 expression was used as an internal control.

### 2.9. Statistical Analyses

The Student’s *t*-test was used to compare the body weight, behavioural changes, immunostained cells, γH2AX foci, and mRNA and miRNA expression by qRT-PCR between the control and irradiated mice. *p* < 0.05 was considered statistically significant. miRNA and mRNA analyses were based on the parameters |log2FC| > 0.585 and *p* < 0.05, which were considered as significantly differential expression of DEseq2.

## 3. Results

### 3.1. Body Weight and Behavioural Changes

**Figure 1:**
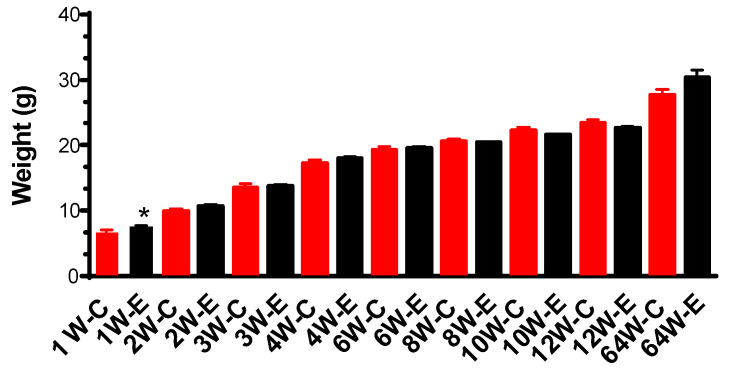
Weight measurement indicates that chronic irradiation with a dose rate of 1.2 mGy/h did not affect weight gain from 2 weeks during irradiation until 64 weeks after the first irradiation started. Animal weight gain increased significantly during the first week of irradiation. The Student’s *t*-test was used to compare the body weight between control and exp groups (1.2 mGy/h) at different time points. * *p* < 0.05. Control: n = 9; Exp (1.2 mGy/h): n = 15. W: week; C: control; E: exp (1.2 mGy/h); 1W-C means control mice in 1 week.

Animal body weight increased one week after γ-irradiation and then did not show any significant difference between the control and irradiated groups from two weeks until 64 weeks (Figure 1).

**Figure 2:**
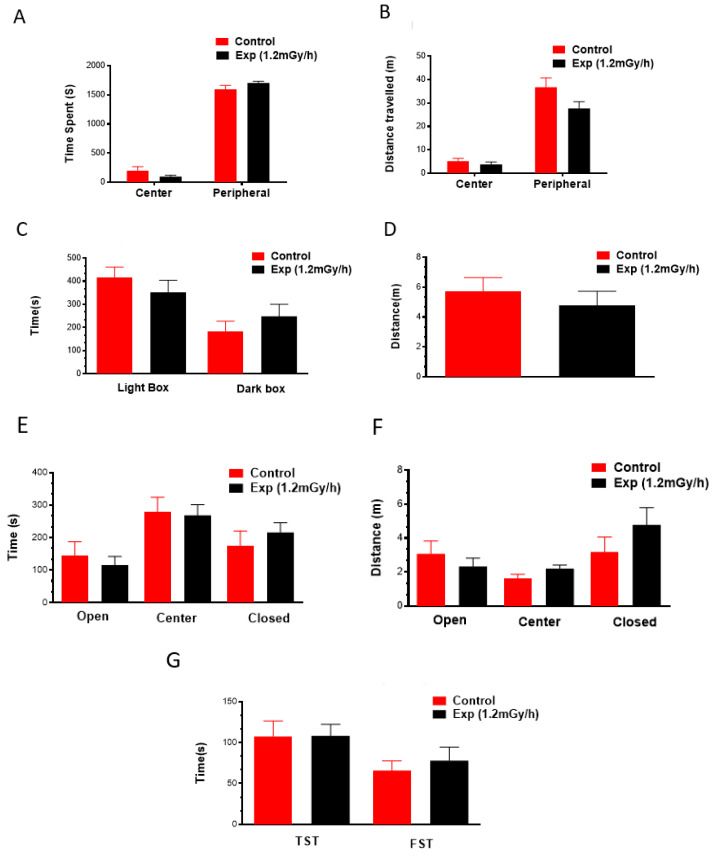
Neurobehavioural tests did not show chronic irradiation-induced anxiety and depression behavioral changes. Time spent (**A**) and distance travelled (**B**) in each area in the open field test; time spent (**C**) in the light and dark box and distance travelled (**D**) in the light box in the light–dark box test; time spent (**E**) and distance travelled (**F**) in three areas in the elevated plus maze; (**G**) time immobile in the tail suspension test and forced swim test. TST: tail suspension test; FST: forced swim test. The Student’s *t*-test was used to compare the data between the control and exp groups (1.2 mGy/h). Control: n = 9; exp group: n = 15.

The open field test showed that the control and the irradiated animals spent almost the same time in the center and peripheral areas (Figure 2A). The distance travelled also showed no differences in these two areas (Figure 2B). In the light–dark box test, the time spent and distance travelled in the light box did not differ between control and experimental groups (Figure 2C,D). Moreover, animals did not exhibit any changes in anxiety-like behaviour in the elevated plus maze, indicated by the time spent and distance travelled in the center, open, and closed arms (Figure 2E,F). Tail suspension and forced swim tests did not show a significant difference in immobile time between the control and the irradiated mice (Figure 2G). All the tests demonstrated that chronic low-dose-rate postnatal γ-irradiation did not induce anxiety-like behaviour, stress, or depression changes in mice.

### 3.2. Immunohistochemistry Examination

**Figure 3:**
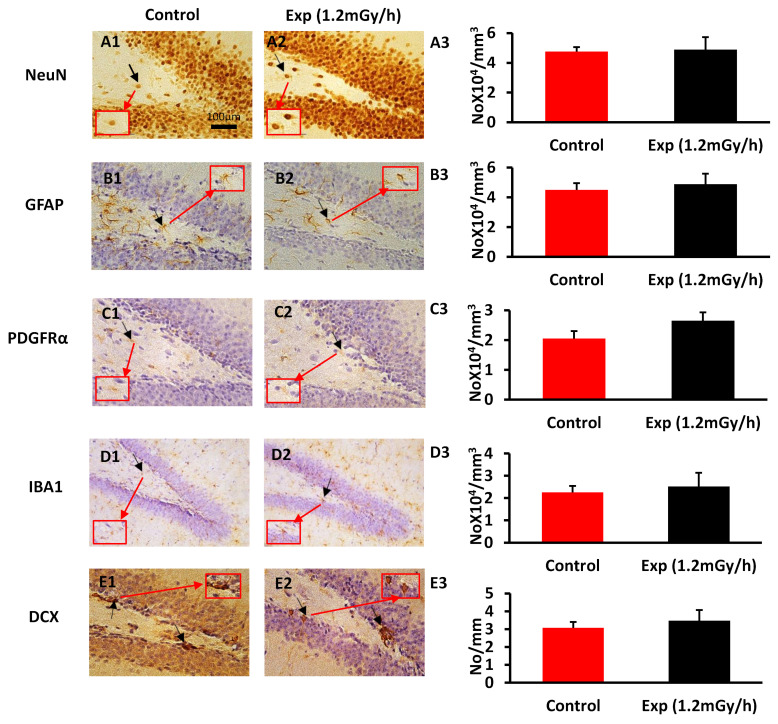

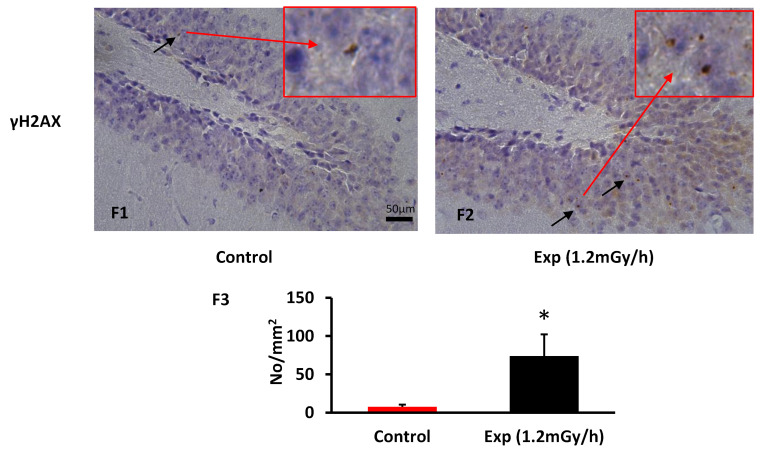
Immunohistochemical staining of the dentate gyrus of the hippocampus in the control and experiment mice. (**A1**–**A3**): NeuN immunopositive mature neurons (arrow); (**B1**–**B3**): GFAP immunopositive astrocytes (arrow); (**C1**–**C3**): PDGFRα immunopositive oligodendrocyte precursor cells (arrow) in the hilus; (**D1**–**D3**): IBA1 immunopositive microglia (arrow) in the hilus and the granule cell layer; (**E1**–**E3**): DCX immunopositive immature neurons (arrow) in the subgranular zone; (**F1**–**F3**): γH2AX immunostaining shows DNA damage foci in the granule cells. Scale bar = 100 μm in (**A1**) applies to (**B1**–**E1**) and (**A2**–**E2**). Scale bar = 50 μm in (**F1**) applies to (**F2**). (**A3**–**F3**): statistical results. * p < 0.05. Control: n = 8; exp group: n = 7.

Immunohistochemical study did not display a significant difference in the number of mature neurons (NeuN) in the hilus, which are not related to the immature neurons (Figure 3A1–A3), astrocytes (GFAP) (Figure 3B1–B3), and oligodendrocyte precursor cells (PDGFRα) (Figure 3C1–C3) in the hilus of the dentate gyrus of the hippocampus, nor the microglia (IBA1) in the hilus and the stratum granulosum (Figure 3D1–D3) between the control and irradiated groups. There was also no change in the number of immature neurons (DCX) in the subgranular zone of the dentate gyrus (Figure 3E1–E3). However, γH2AX immunostaining showed a greatly significant increase in the irradiated mice compared with the control group (Figure 3F1–F3), indicating persistent DNA damage foci even 64 weeks after postnatal irradiation.

### 3.3. mRNA Sequencing and qRT-PCR in the Hippocampus

**Figure 4:**
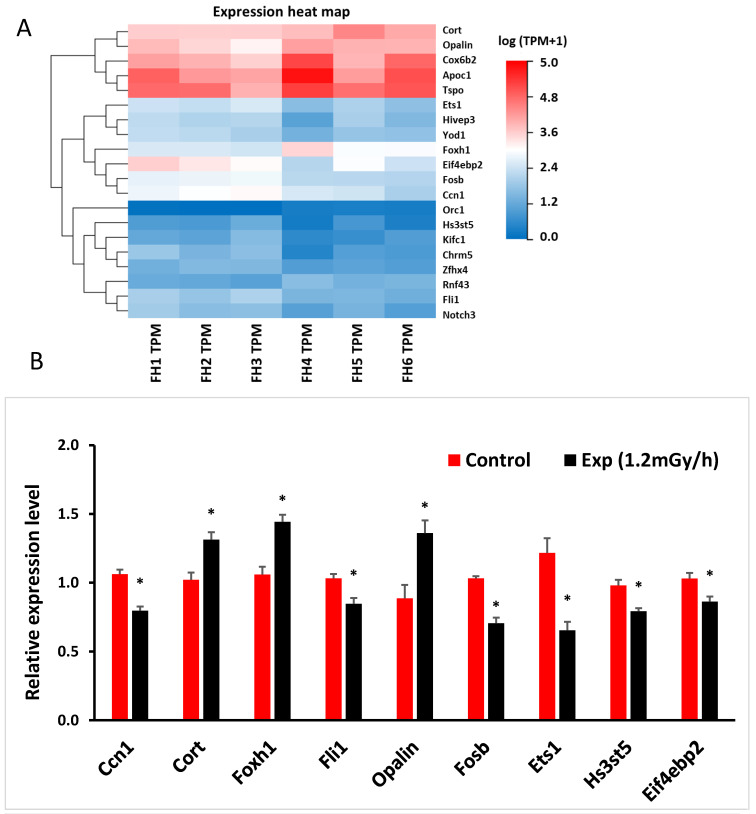
Low-dose-rate irradiation-induced hippocampal mRNA changes: (**A**) Heatmap of mRNA changes from mRNA sequencing in the control and experiment (Exp) mice. (**B**) qRT-PCR indicates a significant down-regulation of *Ccn1*, *Fli1*, *Fosb*, *Ets1*, *Hs3st5,* and *Eif4ebp2* genes, and up-regulation of *Cort*, *Foxh1*, and *Opalin* genes. * *p* < 0.05. Control: n = 3; Exp group: n = 3. FH: female hippocampus; FH1, FH2, FH3 are controls; FH4, FH5, FH6 are exp mice (1.2 mGy/h); TPM: transcript per million.

Based upon a *p*-value of less than 0.05 and a fold change of more than 1.5 between the control and irradiated samples, 107 differentially expressed mRNAs were found in the hippocampus of irradiated mice by the sequencing analysis (Supplementary Figure S1a); some 41 mRNA were upregulated, while 66 were down-regulated. Among differentially expressed mRNAs, we selected 20 genes related to DNA damage or neurological disorders to validate by qRT-PCR (Figure 4B and Figure S1b). The results showed that the expression of cellular communication network factor 1 (*Ccn1*), friend leukaemia integration 1 transcription factor (*Fli1*), protein fosB (*Fosb*), E26 transformation-specific sequence-1 (*Ets1*), heparan sulfate-glucosamine 3-sulfotransferase 5 (*Hs3st5*), and eukaryotic translation initiation factor 4E binding protein 2 (*Eif4ebp2*) were significantly down-regulated in the experimental group. In contrast, corticosterone (*Cort*), forkhead box protein H1 (*Foxh1*), and oligodendrocytic paranodal loop protein (*Opalin*) were up-regulated after γ-irradiation when compared with the control mice (Figure 4B). The expression trend of these genes from qRT-PCR (Figure 4B) was consistent with the mRNA sequencing data, indicated by the heatmap (Figure 4A).

### 3.4. miRNA Sequencing and qRT-PCR in the Hippocampus

**Figure 5:**
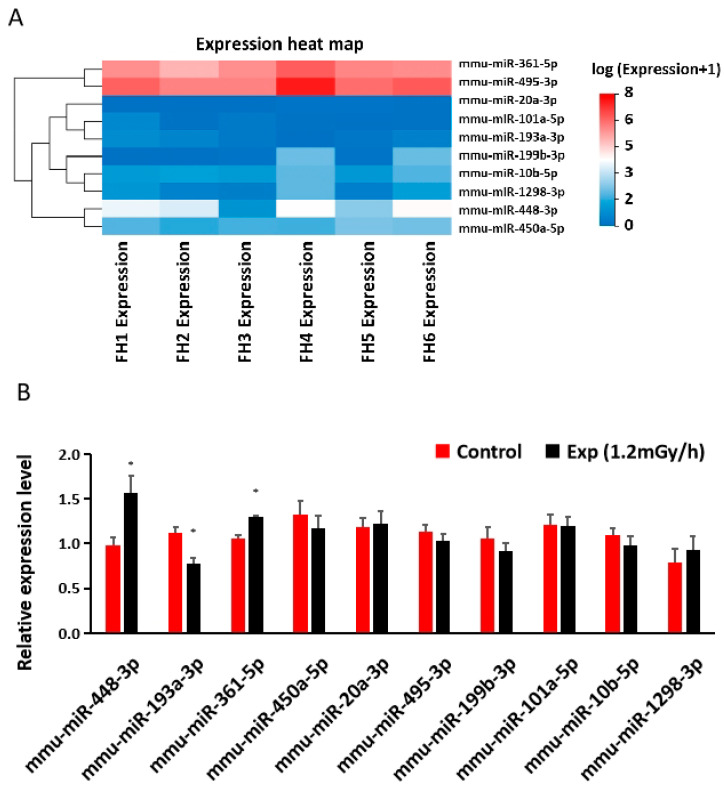
Low-dose-rate irradiation-induced hippocampal miRNA changes: (**A**) Heatmap of miRNA changes from miRNA sequencing in the control and experiment mice; (**B**) qRT-PCR indicates a significant down-regulation of miR-193a-3p and up-regulation of miR-448-3p and miR-361-5p in the irradiated mice (* *p* < 0.05), but no changes for other miRNA investigated (*p* > 0.05). Control: n = 3; Exp group: n = 3. FH: female hippocampus; FH1, FH2, FH3 are controls; FH4, FH5, FH6 are exp (irradiated with 1.2 mGy/h).

Based upon a *p*-value less than 0.05 and a fold change of more than 1.5 between control and irradiated samples, 140 differentially expressed miRNAs were revealed in the hippocampus of irradiated mice by the sequencing analysis (Supplementary Figure S2). We further performed a database search on miRDB and TargetScan on the interaction of these miRNA with the above differentially expressed mRNAs (Figure 4B) and selected 10 miRNAs for validation by qRT-PCR (Figure 5A,B). The expression of two miRNAs, i.e., miR-448-3p and miR-361-5p, increased and was consistent with the miRNA sequencing results (Figure 5A,B). Both qRT-PCR and miRSeq indicated a decreased expression of miR-193a-3p. TargetScan indicated that Fli1 and Hs3st5 might be the targets of miR-448-3p. Eif4ebp2 is the target of miR-361-5p.

### 3.5. mRNA Sequencing and qRT-PCR in the Blood

**Figure 6:**
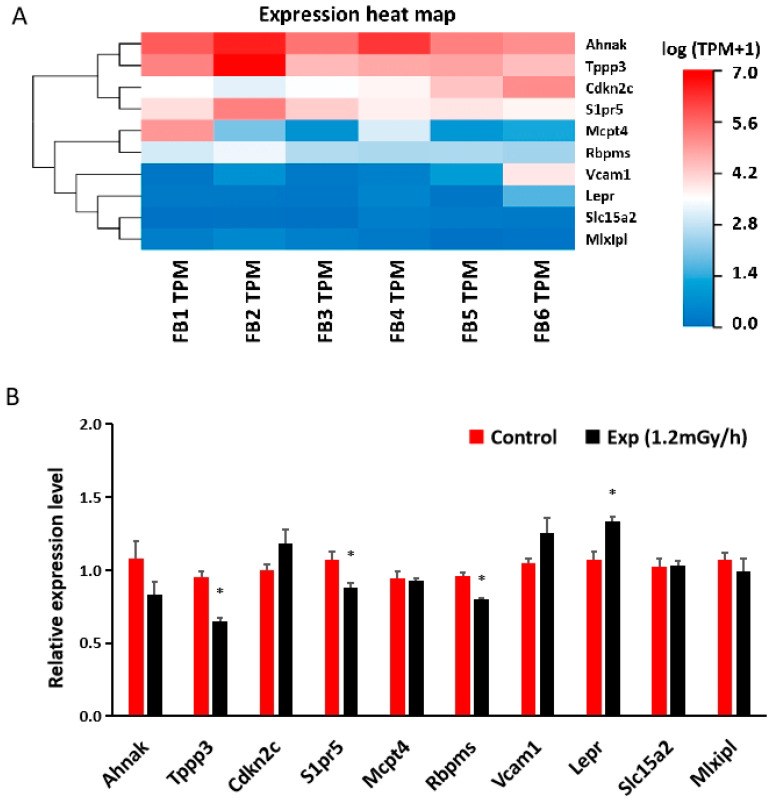
Low-dose-rate irradiation-induced blood mRNA changes: (**A**) Heatmap of mRNA sequencing results in the blood of control and experiment mice; (**B**) qRT-PCR indicates a significant down-regulation of *Tppp3*, *S1pr5*, and *Rbpms* and up-regulation of *Lepr* genes (* *p* < 0.05) but no changes of other mRNAs in the blood of the control and irradiated mice. Control: n = 3; exp group: n = 3. FB: female blood; FB1, FB2, and FB3 are controls; FB4, FB5, and FB6 are exp mice (1.2 mGy/h); TPM: transcript per million.

Based upon *p*-value of less than 0.05 and a fold change of more than 1.5 between control and irradiated samples, 462 differentially expressed mRNAs were found in the blood of irradiated mice by sequencing analysis (Supplementary Figure S3). qRT-PCR study of 10 genes related to DNA damage or neurological disorders showed that the expression of Tubulin polymerization promoting protein family member 3 (*Tppp3*), sphingosine-1-phosphate receptor 5 (*S1pr5*), and RNA-binding protein with multiple splicing (*Rbpms*) was decreased, while the expression of Leptin receptor (*Lepr*) was increased in mouse blood after γ-irradiation (Figure 6A,B).

### 3.6. miRNA Sequencing and qRT-PCR in the Blood

**Figure 7:**
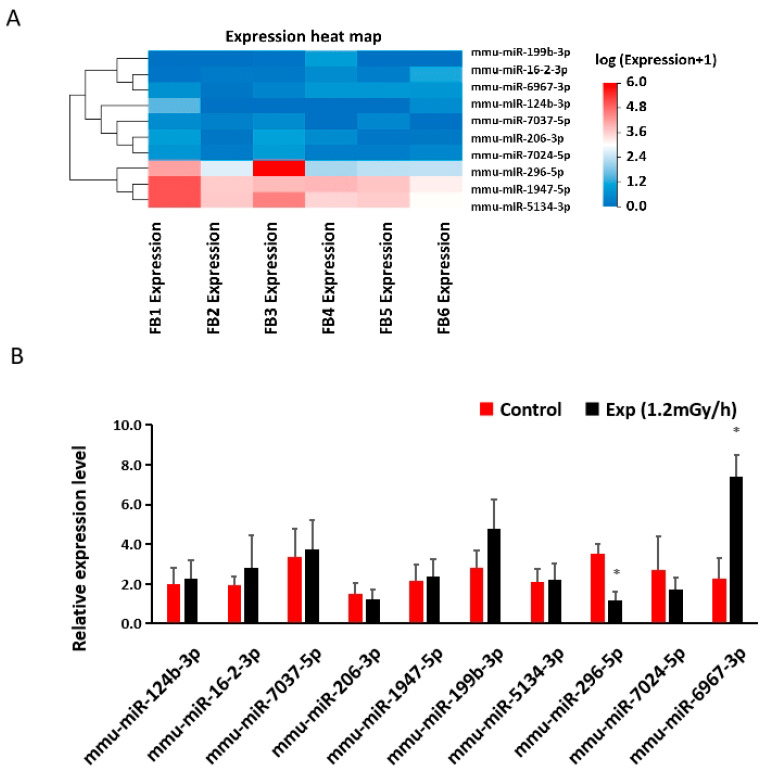
Low-dose-rate irradiation-induced blood miRNA changes: (**A**) Heatmap of miRNA sequencing results in the blood of control and experiment mice; (**B**) qRT-PCR indicates a significant down-regulation of miR-296-5p and up-regulation of miR-6967-3p (* *p* < 0.05) but no changes of other miRNAs in the blood of the control and irradiated mice. The Student’s *t*-test was used to compare the data between control and exp mice (1.2 mGy/h). Control: n = 3; exp group: n = 3. FB: female blood; FB1, FB2, FB3 are controls; FB4, FB5, FB6 are exp (1.2 mGy/h).

Based upon a *p*-value of less than 0.05 and a fold change of more than 1.5 between the control and irradiated samples, sequencing analysis indicated 186 differentially expressed miRNAs in the blood of irradiated mice (Supplementary Figure S4). When 10 miRNAs were selected for validation by qRT-PCR (Figure 7A,B), it was shown that miR-296-5p was down-regulated and miR-6967-3p was up-regulated in blood after γ-irradiation when compared with the control group (Figure 7A,B). TargetScan suggested the interaction of miR-6967-3p and *S1pr5*.

### 3.7. Venn Diagram Analysis of Differentially Expressed mRNAs and miRNAs between Blood and Hippocampus

**Figure 8:**
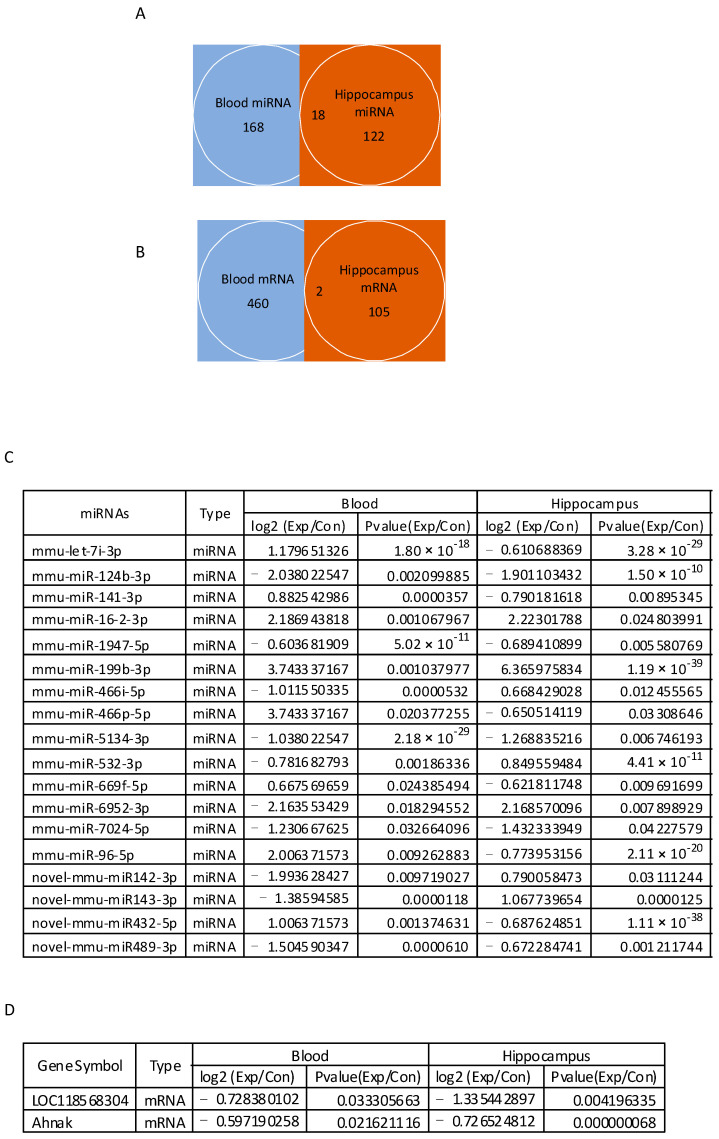
Low-dose-rate irradiation-induced differentially expressed miRNAs and mRNAs in both the blood and hippocampus. (**A**,**B**): Venn diagram of 18 differentially expressed miRNAs (**A**) and two mRNAs (**B**) in both the blood and hippocampus of irradiated mice compared to the control; (**C**) table list of 18 miRNAs differentially expressed in both the blood and hippocampus; (**D**) table list of two mRNAs differentially expressed in both the blood and hippocampus.

A Venn diagram analysis indicates that eighteen miRNAs and two mRNAs are differentially expressed in both the hippocampus and blood, respectively (Figure 8A–D). However, among 18 miRNAs, 11 miRNAs showed an opposite expression pattern in terms of up- or down-regulation in blood, and down- or up-regulation in the hippocampus. Five down-regulated (miR-124b-3p, miR-1947-5p, miR-5134-3p, miR-7024-5p, and novel-miR143-3p) and two up-regulated miRNAs (miR-16-2-3p and miR-199b-3p) plus two down-regulated mRNAs (*Ahnak* and *LOC118568304*) were expressed in both the hippocampus and blood (Figure 8C,D). Among these miRNAs and mRNAs, the expressions of miR-124b-3p, miR-1947-5p, miR-5134-3p, miR-7024-5p, miR-16-2-3p, and miR-199b-3p, as well as *Ahnak*, were further tested in blood by RT-PCR (Figure 6B and Figure 7B). However, the expression of these miRNAs did not show statistically significant changes. Similar results were observed in the hippocampus (Figure 5B).

## 4. Discussion

### 4.1. Main Findings

Based on our previous publication of acute high-dose-rate/dose (3.33 Gy/m, 5 Gy) irradiation-induced pathophysiological changes in the hippocampus [12], this study systemically investigated the effects of a chronic low-dose-rate with cumulative high dose (dose rate: 1.2 mGy/h, total dose: 5 Gy) γ-irradiation from postnatal day 3 to day 180 on animal behaviour, hippocampal cellular change, and miRNA and mRNA expression in the hippocampus and blood in female mice. It showed that the low-dose-rate γ-irradiation with a cumulative dose of 5 Gy did not affect the animal body weight significantly except for an increase in the first week. Neurobehavioural tests, including open field, light–dark box, elevated plus maze, tail suspension, and forced swim tests, did not detect any significant neuropsychiatric changes such as anxiety and depression, which had been found in our previous acutely irradiated mice [12]. NeuN and DCX immunohistochemical staining did not show obvious loss of mature and immature neurons in the dentate gyrus, which was very different from the acute irradiation at postnatal day 3, with 5 Gy showing impairment of neurogenesis and hypoplasia of the low blade of the granule layer [12,18]. IBA1, GFAP, and PDGFRα immunostaining did not demonstrate any inflammatory glial response, which has been observed after acute irradiation [19]. γH2AX immunostaining indicated a significant increase of γH2AX foci in the stratum granulosum of the dentate gyrus, suggesting chronic low-dose-rate irradiation-induced persistent DNA damage, which had also been found after acute irradiation with the same dose [17]. miRNA sequencing and qRT-PCR indicated an increased expression of miR-448-3p and miR-361-5p, but decreased expression of miR-193a-3p in the mouse hippocampus. Meanwhile, mRNA sequencing and qRT-PCR showed a reduced expression of *Ccn1*, *Fli1*, *Fosb*, *Ets1*, *Hs3st5*, and *Eif4ebp1*, but enhanced expression of *Cort*, *Foxh1*, and *Opalin*. Database searching by miRDB and TargetScan predicted that *Fli1* and *Hs3st5* are the targets of miR-448-3p, and *Eif4ebp2* is the target of miR-361-5p. miRNA/mRNA sequencing and qRT-PCR results in the blood showed the increased expression of miR-6967-3p and the decreased expression of its target *S1pr5*. The interactions of these miRNAs and mRNAs may be related to the chronic low-dose-rate radiation-induced persistent DNA damage. Further studies are still needed to clarify the relationship.

### 4.2. Chronic Low-Dose-Rate Irradiation Did Not Induce Abnormal Cellular Changes in the Dentate Gyrus of the Hippocampus and Neuropsychiatric Abnormality

The high-dose-rate acute γ-irradiation (5 Gy) of postnatal day-3 mice induced depression in adults accompanied by hypoplasia, impaired neurogenesis, and cell division in the dentate gyrus [12]. Acute high-dose irradiation induces a complex network of cellular and molecular alterations, e.g., oxidative stress, systemic inflammation, DNA damage, and cell death. However, the effects of low-dose or low-dose-rate γ-irradiation on behavioural, neurological, or brain cellular changes are still controversial. Male adult BALB/c mice showed no significant differences in the immobility times in the forced swim test after receiving 7 days of γ-irradiation at 0.6 mGy/h or 3.0 mGy/h (total doses 0.1 or 0.5 Gy) [20]. Female adult Sprague Dawley (SD) rats with continuously chronic low-dose γ-irradiation for 30 days (dose rates 6 and 20 mGy/h, with a total dose of 0.9 Gy and 3 Gy) had impaired learning memory but no anxiety or depression [21]. The image examination did not reveal any noticeable structural changes in the brain, but rats irradiated with 20 mGy/h had neuronal damage in the hippocampus [21]. Very recently, we found that in prenatally irradiated male B6C3F1 mice (100 mGy/d for 18 days), there were no cellular changes in immature neurons, mature neurons, or glial cells in the dentate gyrus of the hippocampus, but there was a significant reduction in body weight, mass index (BMI) and exploratory behaviour in the open field test [5]. However, when pregnant Wistar rats received γ-irradiation (total cumulative dose of 3 Gy at 0.7 mGy/min) from embryonic day E13 to E16, the adult offspring showed a significant decrease in the numbers of hippocampal pyramidal and granule cells [22]. The diversity may be due to the differences in animal species, sex, age, behaviour test models, and examination methods.

In this study, five behaviour tests were selected. The open field test was designed to examine locomotor activity, anxiety-like behaviour, and the willingness to explore in animals. The light–dark box was used to test the mouse’s innate aversion to bright areas and the spontaneous exploratory behaviour in response to mild stressors, *e.g.,* light. The elevated plus maze was based on the natural aversion of rodents to height, in terms of an unprotected and completely elevated area as an anxiogenic challenge. Based on the number of animals we used, the irradiated mice in this study did not show any stress, depression (evaluated by tail suspension test and forced swim test), anxiety-like behaviour, or abnormal locomotor activity (examined by light–dark box, open files test, and elevated plus maze). We cannot exclude the possibility that increasing the animal numbers, extending the animal survival time, and testing animals at older ages may result in different research findings compared to the present study. Furthermore, only female results were reported in this study; this result may not apply to male mice. Further extensive studies with increasing animal numbers in both male and female mice, animal age groups, different radiation dose rates with cumulative doses, and experimental end-points may provide more convincing results for understanding brain effects of continuous chronic low-dose-rate radiation exposure with cumulative high doses.

### 4.3. Chronic Low-Dose-Rate Irradiation-Induced Persistent DNA Damage Foci

The phosphorylated histone γH2AX has been demonstrated to form foci in nuclei and megabase chromatin domains after DNA lesions on chromosomes. It has been well-recognized as a biomarker for DNA damage from radiation exposure [23,24]. DNA double-strand breaks (DSBs) are the most relevant lesions in the ionizing radiation-induced deleterious effects. The induction of DSBs leads to the phosphorylation of H2AX, which is believed to initiate the DNA damage response [25]. γH2AX has been used as a biodosimetry tool for radiation exposure assessment [24,26,27]. Very recently, Ramadhani et al. used an enzyme-linked immunosorbent assay (ELISA) to quantify γH2AX in male human peripheral blood mononuclear cells (PBMCs) after exposure to different doses of ^60^Co and showed that the ratio of γH2AX/total H2AX increased in a radiation-dose-dependent manner [24]. Chen et al. employed another method of flow cytometry to measure γH2AX fluorescence in male BALB/c mouse blood exposed to X-rays and observed that the fluorescence intensity increased with radiation dose [27]. Mass spectrometry quantification of γH2AX was also developed as an estimation assay in human peripheral blood lymphocytes for low-level exposure to ionizing radiation [28]. Moreover, γH2AX induction was observed in a swine model [29], in patients after abdominal-pelvic and chest CT exams with very low-ionizing radiation exposure (doses of 15.67–63.45 mGy) [30], and in a rhesus macaque (*Macaca mulatta*) model after whole-body radiation exposure (^60^Co) [31]. Our group also showed an extensive induction of γH2AX foci in different brain regions age-dependently at 1 day after 5 Gy γ-irradiation at postnatal days 3, 10, and 21 [17]. These findings consistently support the high-dose-rate irradiation-induced DNA damage and γH2AX foci formation. In the present study, although continuous chronic low-dose-rate postnatal γ-irradiation with a cumulative high dose of 5 Gy did not induce the neurobehavioural changes and cellular changes in the dentate gyrus of the hippocampus, γ-irradiation-induced persistent DNA damage was observed 1 year after the last day of radiation exposure. Whether these persistent DNA foci are involved in the development of neurological or neuropsychiatric disorders such as Alzheimer’s disease or Parkinson’s disease at the late stages of animal life remains to be further investigated.

### 4.4. Chronic Low-Dose-Rate Irradiation Induced miRNA and mRNA Changes in the Hippocampus

Our previous studies have demonstrated that miRNAs play an important role in brain morphological and behavioural changes induced by acute high-dose γ-irradiation in mice [12,19,32]. The γ-radiation-activated miR-43a-5p/*Tia1* pathway in the early life of mice was demonstrated to be related to the pathogenesis of adult depression [12]. The interaction of miR-181b-2-3p and its target SRY-related high-mobility group box transcription factor 21 (*Sox21*) plays a duel role in brain inflammation and the impairment of neurogenesis induced by ionizing radiation through inducing apoptosis in neurogenic zones and activating microglia [19]. These novel findings may provide a new therapeutic way to prevent and inhibit the radiation-induced pathogenesis of depression. In this study, low-dose-rate irradiation induced up-regulation of miR-448-3p and miR-361-5p but down-regulation of miR-193a-3p. miR-448-3p has been poorly studied so far. Very limited literature has indicated its role in the development of cerebral aneurysms [33,34] and cerebral ischemic injury [35] through the interaction with its direct target Kruppel-like transcription factor 5 (*Klf5*) or nuclear factor erythroid 2-related factor 2 (*Nrf2*). In the former [33], miR-448-3p showed an anti-inflammatory effect by reducing *Klf5*, *Mmp2,* and *Mmp5*, which may prevent the low-dose-rate irradiation-induced glial activation. Whereas in the latter [34], up-regulation of miR-448-3p was observed after brain injury. Meanwhile, the observation that down-regulation of miR-448-3p reduced oxidative stress and apoptosis suggested its involvement in chronic low-dose-rate radiation-induced persistent DNA damage or γH2AX foci in the stratum granulosum of the dentate gyrus, as failure to repair DNA damage may lead to apoptosis.

miR-361-5p was proven to promote oxygen–glucose deprivation/re-oxygenation-induced neuronal injury in vitro by regulating sequestosome 1 (Sqstm1) [36]. It was associated with ubiquitin-protein ligase E3 component N-recognin 5 (UBR5), PARP1, and ataxia-telangiectasia mutated interactor (ATMIN) in tumours, which are enriched in DNA damage and repair [37,38,39]. miR-361-5p up-regulation decreased *Ubr5* expression [38], inhibited ATMIN ubiquitination, attenuated the restoration of ATM, and impaired DNA repair [39]. The evidence that the overexpression of miR-361-5p enhanced apoptosis and *Bax* expression but reduced *Bcl-2* [38], and miR-361-5p mimics lessened the cell viability and DNA repair in UV-irradiated 661W cells [40], suggesting its involvement in low-dose-rate irradiation-induced DNA damage. miR-193a-3p induced the accumulation of intracellular reactive oxygen species (ROS) and DNA damage as determined by the level of γH2AX in the glioma cell line by targeting myeloid cell leukaemia 1 (*Mcl-1*) [41]. The down-regulation of miR-193a-3p decreased the chemoresistance and radioresistance of oesophageal squamous cell carcinoma (ESCC) cells via the presenilin 1 (*Psen1*) gene [42]. In the present study, down-regulation of miR-193a-3p may reduce the accumulation of intracellular ROS and antagonize miR-448-3p and miR-361-5p up-regulation-induced DNA damage to produce a beneficial effect.

Combined mRNA sequencing and qRT-PCR results showed the decreased expressions of *Ccn1*, *Fli1*, *Fosb*, *Ets1*, *Hs3st5*, and *Eif4ebp1*, and the up-regulation of *Cort*, *Foxh1*, and *Opalin* in the hippocampus. Database searching by miRDB and TargetScan predicted that *Fli1* and *Hs3st5* are the targets of miR-448-3p. *Eif4ebp2* is the target of miR-361-5p. *Fli-1*, as a member of the ETS transcription factor family, was first identified in Friend murine leukaemia virus-induced erythroleukemias in 1990 by Ben-David et al. [43]. *Fli1* exerts its functions in the development of hematopoietic stem cells, angiogenesis, and vasculogenesis [44]. Abnormal expression of *Fli1* induced different kinds of human cancers and auto-immune diseases [45,46]. *Fli1* has been broadly studied in Ewing’s sarcoma, but its function in γ-radiation-induced injury has been barely investigated. *Fli1* was found to regulate radiotherapy resistance through the PI3K/AKT signalling pathway in nasopharyngeal carcinoma [47]. Overexpression of *Fli1* promoted resistance to radiation exposure in glioblastoma cells [48]. Moreover, other members of the ETS family, e.g., *Erg* and *Ets2*, were also related to the repair of DNA damage [49]. Our results showed the down-regulated expression of *Fli1* in mice exposed to chronic postnatal low-dose-rate radiation. It suggests that as a radiosensitive gene, Fli 1 may serve as a DNA damage marker in radiation research. *Hs3st5* is one of seven heparan sulfate 3-O-sulfotransferase enzymes [50]. It is related to dementia and amyotrophic lateral sclerosis [51]. The aberrations of the *Hs3st5* gene were associated with intellectual disability and microcephaly with pontine and cerebellar hypoplasia [52]. However, no information is available regarding the role of *Hs3st5* in DNA damage. Our study is the first to detect the decreased expression of *Hs3st5* in mice after γ-irradiation. Moreover, both *Fli1* and *Hs3st5* are the predicted targets of miR-448-3p. The interaction of miR-448-3p with *Fli1* or *Hs3st5* might play a crucial role in low-dose-rate γ-radiation-induced DNA damage in mice. *Eif4ebp2* is a member of the eukaryotic translation initiation factor 4E binding protein family. The products of this family bind *Eif4e* and act as negative regulators of mRNA translation. *Eif4ebp2* is highly expressed in the brain and regulates neuronal stem cell renewal and synapse activity through repressing translation initiation [53]. Gene expression analysis showed that the expression level of *Eif4ebp2* was higher in the radiosensitive breast cancer cells when compared to the radioresistant cells [54], suggesting that *Eif4ebp2* might be a biomarker of radiotherapy reaction in breast cancer. However, its function in radiation-induced DNA damage in normal animals has not been studied. The down-regulated *Eif4ebp2* may serve as a biomarker for γ-radiation-induced injury too. Nevertheless, more studies are still needed to confirm the exact role of *Eif4ebp2* in γ-radiation exposure and its underlying mechanisms.

### 4.5. Chronic Low-Dose-Rate Irradiation Induced miRNA and mRNA Changes in the Blood

miRNA and mRNA sequencing of whole blood indicated a total of 186 and 462 differentially expressed miRNAs and mRNAs between the control and experimental groups, respectively. Among those miRNAs and mRNAs validated by qRT-PCR, further bioinformatic analysis revealed a predicted interaction of miR-6967-3p and *S1pr5*. Sphingosine 1-phosphate (*S1p*) and its five-specific high-affinity receptor (*S1pr*) subtypes *S1pr1–5* have significant regulatory effects in normal physiology, brain and cardiac development, inflammation, angiogenesis, vascular permeability, and cancer growth and metastasis [55]. *S1pr1* was the first of this family to be discovered in 1990 [56] and is one of the most widely studied receptors of *S1p*. *S1pr1* and *S1pr5* are expressed by several cell types of the central nervous system, including microglia, which produce pro-inflammatory cytokines and molecules [57], and are closely related to Parkinson’s disease [58]. *S1pr1* and *S1pr5* were transiently induced in C57BL6/J mice after transient middle cerebral artery occlusion for 30 min [59]. However, as *S1pr5* has not been extensively studied and miR-6967-3p is a relatively new miRNA, the role of their interaction in γ-irradiation-induced injury has not been reported.

A Venn analysis of sequencing data between blood and hippocampus indicated that eighteen miRNAs and two mRNAs were differentially expressed after chronic low-dose-rate irradiation. Among them, five down-regulated miRNAs, two up-regulated miRNAs, and two down-regulated mRNAs were expressed in the same pattern in both the hippocampus and blood. However, RT-PCR did not validate those irradiation-induced miRNA and mRNA changes in both the blood and hippocampus. RNA sequencing is a catch-all technique using only one single primer in the reaction. Real-time PCR, which requires two primers from opposite strands, is a more targeted approach for gene expression analysis. Both methods offer sensitive and reliable transcript quantification, but the generated results might be different. More tests are still needed to further confirm if the discrepancy between the two methods is due to the method sensitivity or possibly the false positives in RNA-seq. Nevertheless, a Venn analysis of sequencing data indicated that most radiation-induced blood miRNA or mRNA biomarker changes may not occur in the hippocampus, although they may serve as biomarkers for the changes of corresponding genes in the hippocampus. In other words, some blood biomarker testing may provide information on the γ-irradiation-induced damage to body organs.

## 5. Conclusions

In summary, this study systemically examined the effects of continuously chronic postnatal low-dose-rate γ-irradiation on the neurobehaviour and hippocampal cellular and molecular biological changes in female mice. Compared to our previous study of acute high-dose-rate radiation exposure to P3 mice [12,17,18,19,60], chronic low-dose-rate irradiation with the same cumulative dose of 5Gy significantly diminished the neuropathological changes and subsequent neurocognitive and neuropsychiatric changes. Although this chronic γ-irradiation did not induce any significant changes in body weight, animal neurobehaviours, or hippocampal cellularity, the DNA damage indicated by γH2AX immunostaining was observed at 64 weeks post-irradiation. This non-recoverable DNA damage might be related to the interactions between miR-448-3p with *Fli1* and/or *Hs3st5* or miR-361-5p with *Eif4ebp2* in the hippocampus. The functional significance of the interaction between miR-6967-3p and *S1pr5* in the blood remains to be investigated.

## Figures and Tables

**Table 1:** Primer sequences for mRNA qRT-PCR in hippocampus and blood.

Gene Name	Primer Sequence
Ccn1 F	CGTCCTTGTGGACAACCAGT
Ccn1 R	CATGATGCTTGCGCTTCTCC
Cort F	TGTGAGATGCCAACGAGACC
Cort R	TGTTGTCGGTAGCGAGCATT
Foxh1 F	CAGGCTGAAACTGGCTCAGA
Foxh1 R	AGGAGCTAGAGGGTCCAGTG
Fli1 F	ATCTGAAGGGGCTACGAGGT
Fli1 R	TGACTCTCCGTTCGTTGGTG
Opalin	GATGAGCCCCGTATGTCCTG
Opalin	GCCTGTCCTAACTTGTGCCA
Fosb F	CCTTCAGTCCCAAAGACGAGT
Fosb R	GGGTGGGGTTTGGGATTAGG
Ets1	CGGTCAGCGGGAATTTGAGA
Ets1	ATCTCCTGGCCACCTCATCT
Hs3st5	GGCGTGTCTGAATGTAGGCT
Hs3st5	CTCCTTCCCCTCTAGCACCT
Eif4ebp2 F	AGCAGAAGTGCCAACACCTT
Eif4ebp2 R	GATGTGGAAAATGGCCCGGT
Zfhx4 F	TAAGGCTGAGACTTGGCTGC
Zfhx4 R	CCCTGTCAGGCTCTATCCCT
Yod1 F	TTTGACCCCATTTCCCCAGT
Yod1 R	TAGGTTGGCCAGTAACCCCT
Hivep3 F	CCCACCATCCCCACTGAAAG
Hivep3 R	GGCAACCCGGGCTCCTTTAT
Chrm5 F	GCTTGTCAAGGTGCAAGGTC
Chrm5 R	GTCCCTGCTGTTCTTCACAGA
Cox6b2 F	TTTTCTCCCGTGCTCTTGGG
Cox6b2 R	AGTACTCGCAGGGTTGTGTG
Tspo F	CTTGGGTCTCTACACTGGTC
Tspo R	AGACTTTATTTAGCTTTAAAACACC
Notch3 F	CTCTCCCTGCCTCAACTTCC
Notch3 R	CTCCCAAATGTCCCCTGACC
Kifc1 F	GAGCCTGCAAAGAAACGGAC
Kifc1 R	TATATGCCACCTACTGCCAGG
Orc1 F	AAGTGTTGGAGAAGTTACGGT
Orc1 R	GACCAACCCACCAGGGATTT
Apoc1 F	GGGCGGTGGTGAATACTAGC
Apoc1 R	TGGCTACGACCACAATCAGG
Rnf43 F	AATTTGTTTCATCCCCGTGCC
Rnf43 R	CTCCCATCGTCACTGCGAAT
Ahnak F	CAGTCAGCACTGCGACCTC
Ahnak R	TTTGCAGGACTCTGCTCAGG
Tppp3 F	TAGAAGCCGGGTGGCATGG
Tppp3 R	GTTCTTTGTGGGAGCCCGTA
Cdkn2c F	CCGGCACAGTACCTTCAGAG
Cdkn2c R	AGCTCAGGCTCTTCACTGCAA
S1pr5 F	ACACCAAATGCCCAGCTTAC
S1pr5 R	AAGTCTCCTGTAACCGGCAC
Mcpt4 F	AGAATCTCTCTCCAAGCTGT
Mcpt4 R	GTAAGGGCGAGAATGTGGTC
Rbpms F	ATTGCCTCAAGAGGAGCAGG
Rbpms R	GGGCGGTCTATCTGACATGG
Vcam1 F	ACTTTCTAATTCATGGTAGAATGGC
Vcam1 R	CAATGAAGAAACAGGTCCCCG
Lepr F	TGATAATGGTGTGACGGTTGC
Lepr R	GGAAGCTTTCACACACTGAA
Slc15a2 F	CAGGGAACGAGCTTGGGAAT
Slc15a2 R	GCAGTTGTCTGGGGAAAGGA
Mlxipl F	CCTGAGCATCTGCAGCCTC
Mlxipl R	ATGACAGCCTCAGGTTTCCG
Gapdh F	ACCACAGTCCATGCCATCAC
Gapdh R	TCCACCACCCTGTTGCTGTA

**Table 2:** miRNA sequences for qRT-PCR in hippocampus and blood.

miRNA	Primer Sequence
mmu-miR-448-3p	TTGCATATGTAGGATGTCCCAT
mmu-miR-193a-3p	AACTGGCCTACAAAGTCCCAGT
mmu-miR-361-5p	TTATCAGAATCTCCAGGGGTAC
mmu-miR-450-5p	CGTTTTGCGATGTGTTCCTAAT
mmu-miR-20a-3p	ACTGCATTACGAGCACTTAAAG
mmu-miR-495-3p	AAACAAACATGGTGCACTTCTT
mmu-miR-199b-3p	ACAGTAGTCTGCACATTGGTTA
mmu-miR-101a-5p	TCAGTTATCACAGTGCTGATGC
mmu-miR-10b-5p	TACCCTGTAGAACCGAATTTGTG
mmu-miR-1298-3p	CATCTGGGCAACTGATTGAACT
mmu-miR-124b-3p	TCAAGGTCCGCTGTGAACACGG
mmu-miR-16-2-3p	GACCAATATTATTGTGCTGCTTT
mmu-miR-7037-5p	AAGGTGGCCACAGGAGATCATGGT
mmu-miR-206-3p	TGGAATGTAAGGAAGTGTGTGG
mmu-miR-1947-5p	AGGACGAGCTAGCTGAGTGCTG
mmu-miR-199b-3p	ACAGTAGTCTGCACATTGGTTA
mmu-miR-5134-3p	ACGGGTGGCCCTCTTTCTGCAG
mmu-miR-296-5p	AGGGCCCCCCCTCAATCCTGT
mmu-miR-7024-5p	TTGGGGGATGGGTTGCTTGGC
mmu-miR-6967-3p	TCATCTTTATCTCTCCCCAG
mmu-miR-68	GCTGTACTGACTTGATGAAAGTAC

## Data Availability

The data presented in this study are available on request from the corresponding author.

## Supplementary Materials

The following supporting information can be downloaded at: https://www.mdpi.com/article/10.3390/cells13201705/s1, Figure S1a: Heatmap of mRNA sequencing data in female hippocampus; Figure S1b: The expression of mRNA in female hippocampus examined by qRT-PCR; Figure S2: Heatmap of miRNA sequencing data in female hippocampus; Figure S3: Heatmap of mRNA sequencing data in female blood; Figure S4: Heatmap of miRNA sequencing data in female blood.
