# Supplementary material for: Chronic Low-Dose-Rate Radiation-Induced Persistent DNA Damage and miRNA/mRNA Expression Changes in Mouse Hippocampus and Blood
Source: Cells. 2024 Oct 15;13(20):1705. doi: 10.3390/cells13201705 (PMC11505968; doi:10.3390/cells13201705)
Supplement: Supplementary file 1 [file cells-13-01705-s001.zip › cells-3204746-supplementary.pdf]

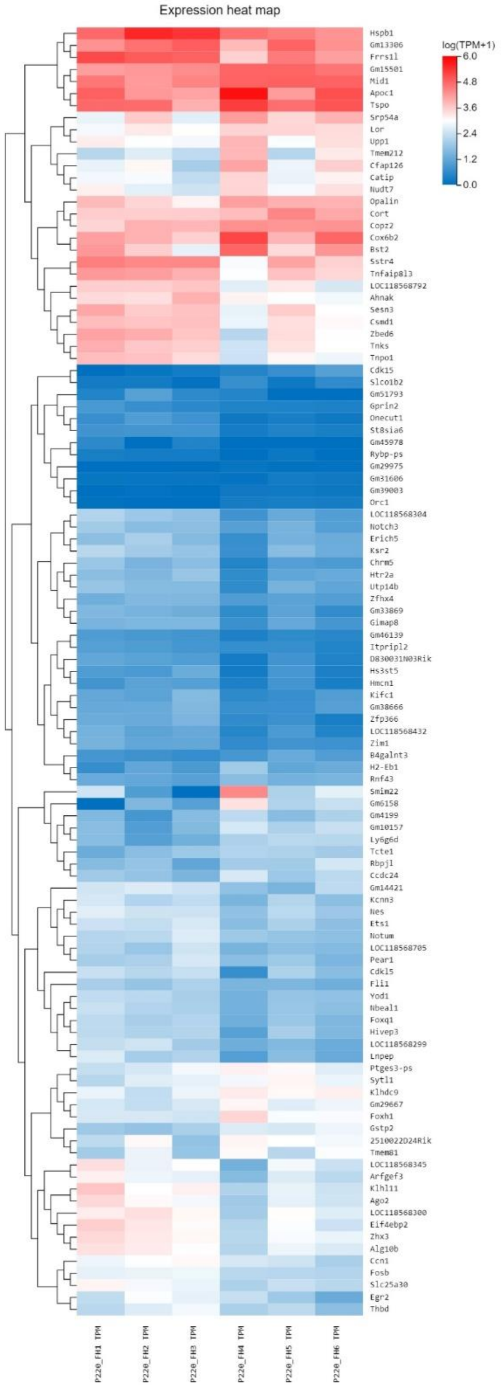

Figure S1a. Heatmap of mRNA sequencing data in female hippocampus.

Figure S1b. The expression of mRNA in female hippocampus examined by qRT-PCR.

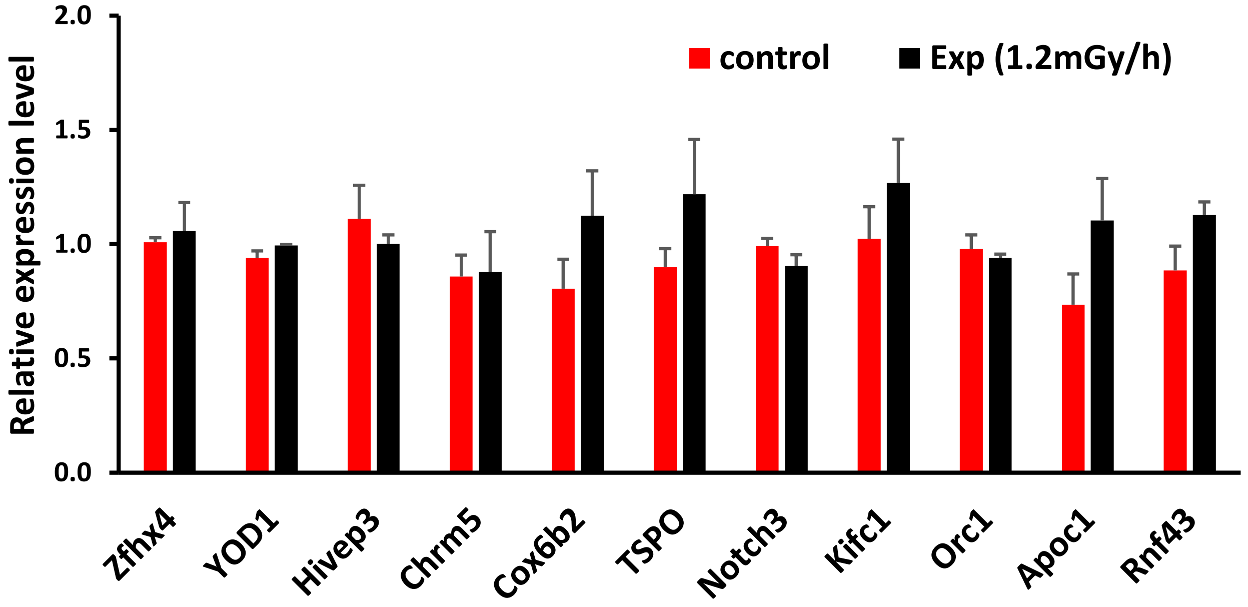

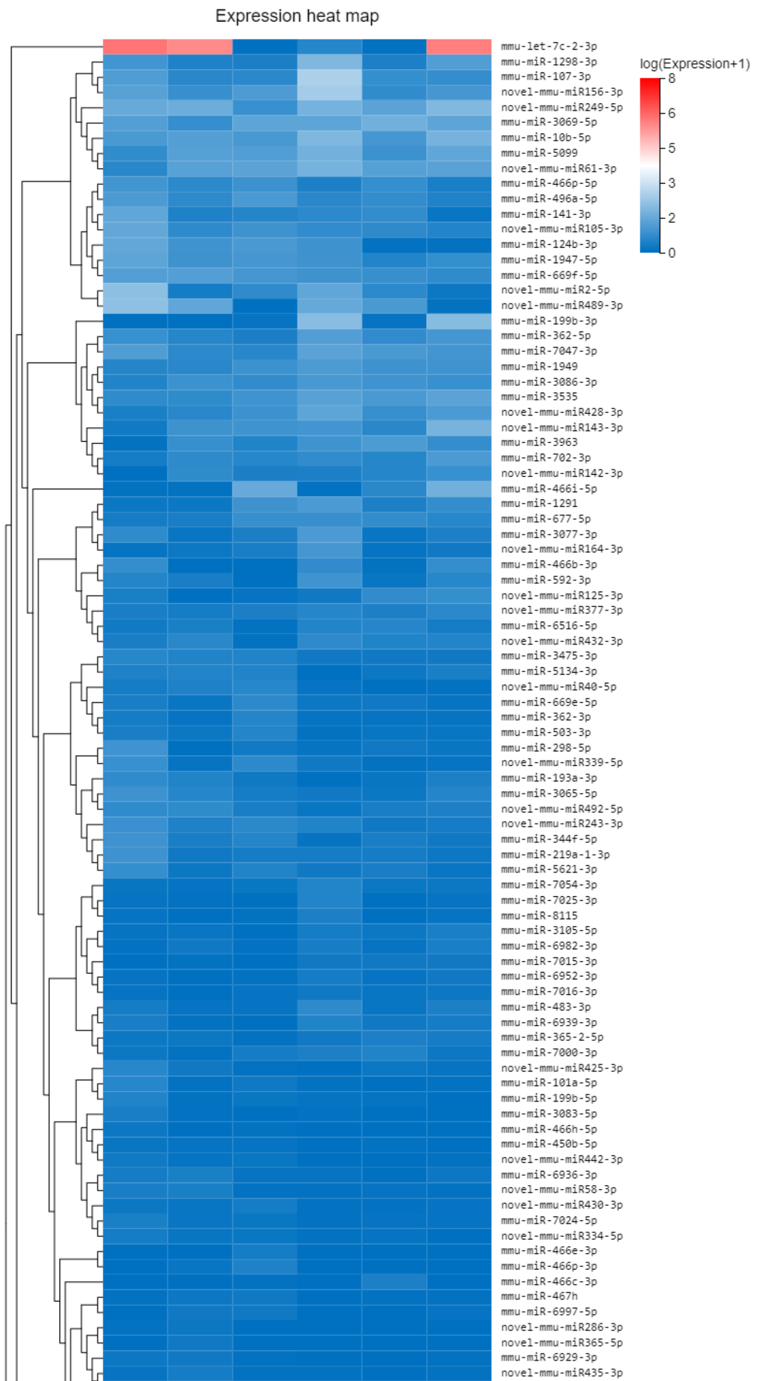

Figure S2. Heatmap of miRNA sequencing data in female hippocampus.

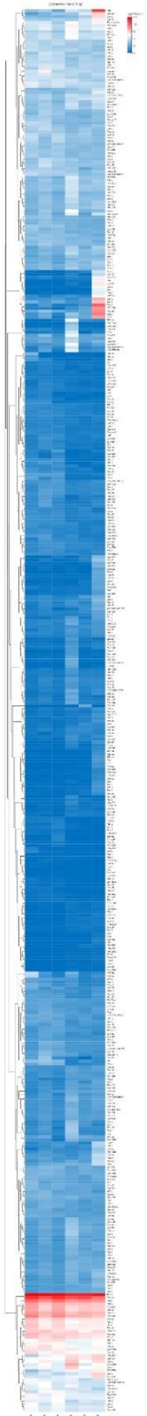

Figure S3. Heatmap of mRNA sequencing data in female blood.

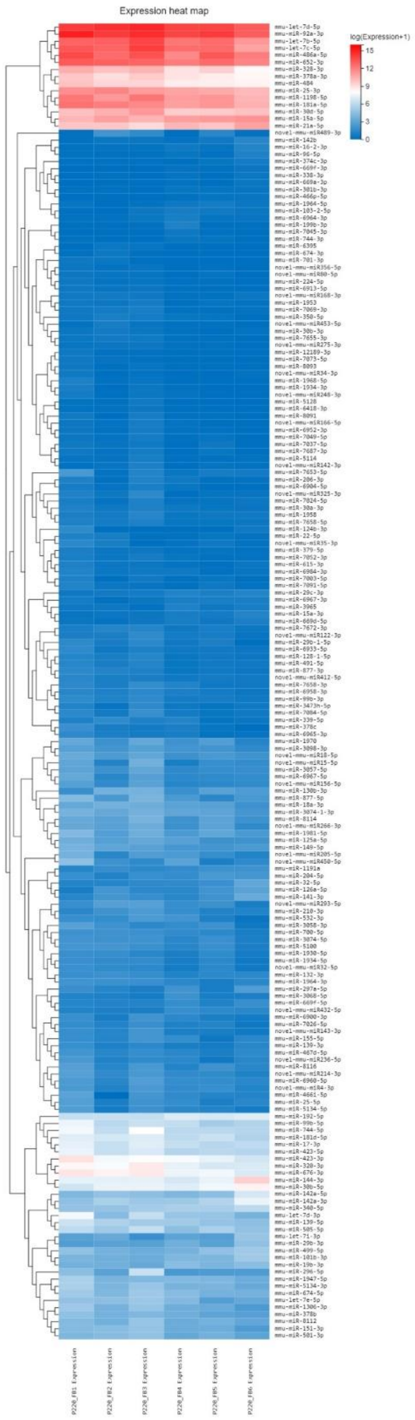

Figure S4. Heatmap of miRNA sequencing data in female blood.
